# Supplementary material for: Emergence of topological superconductivity in doped topological Dirac semimetals under symmetry-lowering lattice distortions
Source: Sci Rep. 2021 Sep 17;11:18539. doi: 10.1038/s41598-021-97982-1 (PMC8448755; doi:10.1038/s41598-021-97982-1)
Supplement: Supplementary file 1 — Supplementary Information. [file 41598_2021_97982_MOESM1_ESM.pdf]

# Emergence of Topological Superconductivity in Doped Topological Dirac Semimetals under Symmetry-Lowering Lattice Distortions

Sangmo Cheon,[1,2,3,4] Ki Hoon Lee,[1,2,5] Suk Bum Chung,[1,2,6,7] and Bohm-Jung Yang[1,2,8]

[1] *Department of Physics and Astronomy, Seoul National University, Seoul 08826, Korea*
[2] *Center for Correlated Electron Systems, Institute for Basic Science (IBS), Seoul 08826, Korea*
[3] *Department of Physics and Research Institute for Natural Science, Hanyang University, Seoul 04763, Korea*
[4] *Institute for High Pressure, Hanyang University, Seoul 04763, Korea*
[5] *Department of Physics, Incheon National University, Incheon, 22012, Republic of Korea*
[6] *Department of Physics, University of Seoul, Seoul, 02504, Korea*
[7] *Natural Science Research Institute, University of Seoul, Seoul, 02504, Korea*
[8] *Center for Theoretical Physics (CTP), Seoul National University, Seoul 08826, Korea*

## CONTENTS

S1. Dirac semimetal 2
  S1.1. Basis and $D_{4h}$ point group elements 2
  S1.2. Ellipsoidal coordinates for Fermi surfaces 2

S2. Distorted Dirac semimetal 2
  S2.1. Review of experimental results 2
  S2.2. Classification of symmetry-lowering lattice distortions 3
  S2.3. Lattice Hamiltonian under lattice distortions 4
  S2.4. Enhancement of DOS at Fermi surface 5

S3. Superconducting Gap Structure 7
  S3.1. Under $n_1$ and $n_2$ type lattice distortions 7
    S3.1.1. $\Delta_1$ and $\Delta_1'$ phases 7
    S3.1.2. $\Delta_2$ and $\Delta_3$ phases 7
    S3.1.3. $\Delta_{41}$ and $\Delta_{42}$ phases 8
  S3.2. Under $n_3$ type lattice distortions 9

S4. Stability of nodal structures 10
  S4.1. Chiral winding number 10
  S4.2. Mirror chiral winding number 10
  S4.3. Accidental point nodes in $\Delta_{41}$ and $\Delta_{42}$ phases 11

S5. Superconducting gap equations 12

S6. Numerical parameters 14

References 14

# S1. DIRAC SEMIMETAL

## S1.1. Basis and $D_{4h}$ point group elements

For convenience, we adopt the following basis set, which is known to describe the low energy effective Hamiltonian of $Cd_2As_3$ [1, 2]:

$$|1,\uparrow\rangle = \left|P_{J=\frac{3}{2}}, 3/2\right\rangle, \quad |1,\downarrow\rangle = \left|P_{J=\frac{3}{2}}, -3/2\right\rangle, \quad |2,\uparrow\rangle = \left|S_{J=\frac{1}{2}}, 1/2\right\rangle, \quad |2,\downarrow\rangle = \left|S_{J=\frac{1}{2}}, -1/2\right\rangle, \tag{1}$$

where $J$ is the total angular momentum. In this basis, the matrix representations for three generators $P$, $C_{4z}$ and $C_{2x}$ of $D_{4h}$ group are obtained. Because the inversion operator does not flip the spin, the inversion operator depends only on the parity of the $s$- and $p$-orbitals. We choose $P = -\sigma_z$ without loss of generality. Using $R(\theta) = e^{-i\theta\mathbf{n}\cdot\mathbf{J}}$, two rotation operators are given by $C_{4z} = \exp[-i\frac{\pi}{2}s_z - i\frac{\pi}{4}\sigma_z s_z] = (-\sigma_z - is_z)/\sqrt{2}$ and $C_{2x} = i\sigma_z s_x$. The other group elements can be obtained from these group generators, which are summarized in Table S1.

| Operators | Representations |
|---|---|
| $P$ | $-\sigma_z$ |
| $C_{4z}$ | $\exp[-i\frac{\pi}{2}s_z - i\frac{\pi}{4}\sigma_z s_z]$ |
| $C_{2x}$ | $i\sigma_z s_x$ |
| $C_{2z} = C_{4z}^2$ | $i\sigma_z s_z$ |
| $M_{xy} = PC_{2z}$ | $-is_z$ |
| $M_{yz} = PC_{2x}$ | $-is_x$ |
| $M_{zx} = PC_{2x}C_{2z}$ | $-i\sigma_z s_y$ |
| $M_{(110)} = PC_{2x}C_{4z}^{-1}$ | $i(\sigma_z s_x - s_y)/\sqrt{2}$ |
| $M_{(1\bar{1}0)} = PC_{2x}C_{4z}$ | $i(\sigma_z s_x + s_y)/\sqrt{2}$ |

Table S1. Symmetry operators of $D_{4h}$ group. Each mirror operator's subscript represents the corresponding mirror plane by using either Cartesian coordinates or Miller indices.

## S1.2. Ellipsoidal coordinates for Fermi surfaces

When the chemical potential $\mu$ is not large enough, the Fermi surfaces for the low-energy effective Dirac Hamiltonian becomes the two-dimensional surface of an ellipsoid and the equation of the Fermi surface is given by

$$\left[\frac{k_x}{1/v}\right]^2 + \left[\frac{k_y}{1/v}\right]^2 + \left[\frac{k_z \mp k_0}{1/v_z}\right]^2 = \mu^2. \tag{2}$$

This Fermi surface can be represented by the following ellipsoidal coordinates $(r, \theta, \phi)$:

$$k_x = \frac{r}{v}\sin\theta\cos\phi,\ k_y = \frac{r}{v}\sin\theta\sin\phi,\ k_z = \frac{r}{v_z}\cos\theta \pm k_0, \tag{3}$$

where $\theta \in [0, \pi]$ is an azimuthal angle and $\phi \in [0, 2\pi]$ is a polar angle.

# S2. DISTORTED DIRAC SEMIMETAL

## S2.1. Review of experimental results

Recently, pressure-induced superconductivity was reported in $Cd_3As_2$ [3–5] and $Au_2Pb$ [6–10]. In the absence of lattice distortions, both materials share common tetragonal crystal system with $D_{4h}$ point group symmetry and show no superconductivity at low temperature. However, under pressure or cooling, both materials showed superconductivity after the structural phase transition. After the structural phase transition, $Cd_3As_2$ becomes a monoclinic lattice having $C_{2h}$ point group symmetry and $Au_2Pb$ becomes an orthorhombic lattice having $D_{2h}$ point group symmetry. Thus, inversion symmetry (IS) is preserved even under lattice distortions. In addition, the amount of the lattice distortion is not large because the changes of lattice constants are small. Now, we summarize the experimental results focusing on the symmetry-lowering pattern and the observation of unconventional superconductivity.

Let us discuss $Cd_3As_2$ [3–5]. First, it does not show any superconductivity in ambient pressure but becomes superconducting at 1.8 K under pressure ($\approx$ 8.5 GPa) [4]. The structural phase transition occurs at 2.4 GPa from a tetragonal lattice with $D_{4h}$ symmetry to a monoclinic lattice with $C_{2h}$ symmetry. The space group of the undistorted lattice is $I4_1/acd$ (No. 142) and the space group of the distorted lattice is $P2_1/c$ (No. 14). After the structural phase transition, the beta angle of the original tetragonal structure is shifted from $\beta = 90°$ to $\beta = 98°$. Second, the $T_c$ increases from 1.8 K to 4.0 K as the pressure increases to 21.3 GPa. Third, the change of volume is not large. Before the structural phase transition, the unit cell of the tetragonal lattice is a reconstructed $2 \times 2 \times 4$ supercell with a total volume $V \approx 4058$ Å$^3$. After the structural phase transition, the total volume of sixteen unit cells in the monoclinic lattice is $V \approx 3636$ Å$^3$, which is 90% of the original volume. Because the volume change is not large, the strength of lattice distortion can be regarded a weak perturbation. Finally, the observed superconductivity is reported as unconventional [3, 4]. The transport data under magnetic fields showed anomalous behaviors unexplainable by the usual BCS $s$-wave superconductivity. The unconventional zero-bias conductance peak (ZBCP) and double conductance peaks symmetric around zero bias shown in the point-contact measurements are interpreted as a possible Majorana surface state.

Now, let us discuss $Au_2Pb$ [6–10]. First, it exhibits superconductivity at $T_c \approx 1.2$ K after a structural phase transition under cooling [6–10]. At room temperature, $Au_2Pb$ is in the cubic Laves phase with cubic symmetry ($O_h$) with lattice constants $a = b = c = 7.90$ Å. This cubic symmetry has three fourfold symmetries ($C_{4x}, C_{4y}, C_{4z}$) around the high symmetric axes ($x$, $y$, $z$ axes), which protect three pairs of Dirac points that are located on the high symmetric axes ($k_x$, $k_y$, and $k_z$ axes) in BZ. Under cooling, several structural phase transitions occur at $T_1 = 97$ K, $T_2 = 51$ K, and $T_3 = 40$ K. Even though the intermediate lattice structures are not clearly studied, below $T_3 = 40$ K the crystal structure becomes orthorhombic with point group symmetry $D_{2h}$. The measured the lattice constants are $a = 5.58$ Å, $b = 11.19$ Å, and $c = 7.90$ Å. Note that in the reference [6], the axes are differently chosen so that $a = 7.90$ Å, $b = 5.58$ Å, and $c = 11.19$ Å. For the consistency with our work, we choose the twofold rotation axis of orthorhombic lattice as $z$-axis. The space group of undistorted lattice is $Fd\bar{3}m$ (No. 227) and the space group of distorted lattice is $Pbcn$ (No. 60). Second, $T_c$ reaches 4 K at 5 GPa, then decreases with further compression [10]. Third, the local pressure from the point contact increases the $T_c$ [8]. The measured $T_c$ using a hard tip is higher than the measured $T_c$ using a soft tip. Fourth, the change of volume is negligible. The volume of unit cell $V \approx 493$ Å$^3$ is nearly same before and after structural phase transition, which means that the strength of the lattice distortion itself is not large. Finally, the superconductivity in $Au_2Pb$ is also unconventional. The empirical transport data shows usual longitudinal linear magenetoresistivity (MR) behavior, which is not described by the conventional BCS theory [6]. Note that the calculated band structure in the low temperature phase for $Au_2Pb$ has a Fermi surface having electron and hole pockets [6, 8], which seem to provide metallic Fermi surface and conventional BCS pairing [9]. However, the interplay between quadratic bands and linear bands from the DSM is not well-understood due to the complexity, which is beyond this work. Instead, we will focus the two Dirac points and influence of the symmetry-lowering lattice distortion.

### S2.2. Classification of symmetry-lowering lattice distortions

In the minimal four-band model, the summery-breaking lattice distortion terms can be described by the following $4 \times 4$ matrix:

$$H_{\text{pert}} = \sum_i d_i(\mathbf{k})\Gamma_i, \tag{4}$$

where $\Gamma_i = s_j\sigma_k$ and $d_i(\mathbf{k})$ is a real-valued function of momentum. Here, $s_j$ and $\sigma_k$ are Pauli matrices for spin and orbital degrees of freedom in the spin $(\uparrow, \downarrow)$ and the orbital $(1, 2)$ spaces, respectively. By time-reversal symmetry (TRS) and inversion symmetry (IS), the form of $d_i$ and $\Gamma_i$ are restricted. First, as discussed in the main text, TRS and IS allow only six gamma matrices are allowed, which are $\Gamma_0$, $\Gamma_1$, $\Gamma_2$, $\Gamma_3$, $\Gamma_4$, and $\Gamma_5$. Also, the constraint for coefficient function can be easily found. Because the $\Gamma_1, \Gamma_2, \Gamma_3$, and $\Gamma_4$ have odd parities under $T$ and $P$, the coefficient functions $d_1(\mathbf{k})$, $d_2(\mathbf{k})$, $d_3(\mathbf{k})$, and $d_4(\mathbf{k})$ should be a function of momenta with only odd powers. Similarly, the coefficient functions $d_0(\mathbf{k})$ and $d_5(\mathbf{k})$ should be a function of momenta with only even powers. Therefore, the form of allowed lattice term is either $k^{\text{odd}}\,\Gamma_{1,2,3,4}$ or $k^{\text{even}}\,\Gamma_{0,5}$ types.

| | $T$ | $P$ | Class |
|---|---|---|---|
| $k^{\text{odd}}$ | $-$ | $-$ | $A_{1u}, A_{2u}, B_{1u}, B_{2u}, E_u$ |
| $k^{\text{even}}$ | $+$ | $+$ | $A_{1g}, A_{2g}, B_{1g}, B_{2g}, E_g$ |
| $\Gamma_{1,2,3,4}$ | $-$ | $-$ | $B_{1u}, B_{2u}, E_g$ |
| $\Gamma_{0,5}$ | $+$ | $+$ | $A_{1g}$ |
| $k^{\text{odd}}\,\Gamma_{1,2,3,4}$ | $+$ | $+$ | $A_{1g}, A_{2g}, B_{1g}, B_{2g}, E_g$ |
| $k^{\text{even}}\,\Gamma_{0,5}$ | $+$ | $+$ | $A_{1g}, A_{2g}, B_{1g}, B_{2g}, E_g$ |

Table S2. Group theoretical classification of momenta, gamma matrices, and their products. The $TP$ symmetry allows only two types of lattice distortions, either $k^{\text{odd}}\Gamma_{1,2,3,4}$ or $k^{\text{even}}\Gamma_{0,5}$. Thus, resultant perturbation terms are included in the $A_{1g}, A_{2g}, B_{1g}, B_{2g}, E_g$.

| Type | Remaining subgroup | Linear | Quadratic | IR |
|---|---|---|---|---|
| $n_1$ | $D_{2h}$ | $k_z\Gamma_3, k_x\Gamma_1 - k_y\Gamma_2$ | $(k_x^2 - k_y^2)\Gamma_0, (k_x^2 - k_y^2)\Gamma_5$ | $B_{1g}$ |
| $n_2$ | $D'_{2h}$ | $k_z\Gamma_4, k_x\Gamma_2 + k_y\Gamma_1$ | $k_xk_y\Gamma_0, k_xk_y\Gamma_5$ | $B_{2g}$ |
| $(n_3, n_4)$ | $(C_{2h(x)}, C_{2h(y)})$ | $(k_z\Gamma_2, k_z\Gamma_3)$ , $(k_y\Gamma_3, k_x\Gamma_3)$ | $(k_yk_z\Gamma_0, k_xk_z\Gamma_0), (k_yk_z\Gamma_5, k_xk_z\Gamma_5)$ | $E_g$ |
| $(n_5, n_6)$ | $(C_{2h(110)}, C_{2h(1\bar{1}0)})$ | $(k_x\Gamma_4, k_y\Gamma_4)$ | - | $E_g$ |

Table S3. All possible symmetry-lowering lattice distortions up to quadratic order. Symmetry-lowering lattice distortions are classified according to the irreducible representation (IR) of $D_{4h}$ point group. $n_1$ and $n_2$ types belong to the $B_{1g}$ and $B_{2g}$ irreducible representations of $D_{4h}$, respectively, while $n_3$, $n_4$, $n_5$, and $n_6$ types belong to the same two-dimensional $E_g$ irreducible representation. $(110)$ and $(1\bar{1}0)$ are the Miller indices for directions.

Furthermore, using the group theoretical analysis, possible symmetry-lowering lattice distortions can be classified according to the irreducible representation of $D_{4h}$ group, which is summarized in Table S2. $k_x$ and $k_y$ are in $E_u$ class, and $k_z$ is in $A_{2u}$ class. $\Gamma_0$ and $\Gamma_5$ are in $A_{1g}$ class, $\Gamma_1$ and $\Gamma_2$ are in $E_u$ class. $\Gamma_3$ is in $B_{2u}$ class and $\Gamma_4$ in $B_{1u}$ class. Hence $k^{\text{odd}}\ \Gamma_{1,2,3,4}$ and $k^{\text{even}}\ \Gamma_{0,5}$ types are included in $A_{1g}, A_{2g}, B_{1g}, B_{2g}, E_g$. Because the trivial class $A_{1g}$ does not break any crystal symmetry, the possible symmetry-lowering lattice distortions can be categorized into $A_{2g}, B_{1g}, B_{2g}, E_g$ classes. Each lattice distortion breaks $D_{4h}$ point group symmetry into its subgroup symmetry: $A_{2g}$ type gives $C_{4h}$. $B_{1g}$ and $B_{2g}$ types give $D_{2h}$ and $D'_{2h}$, respectively. $E_u$ types gives $C_{2h}$. Because $D_{2h}$, $D'_{2h}$ and $C_{2h}$ are reported in $Cd_3As_2$ and $Au_2Pb$, we consider these types of lattice distortions as discussed in the main test.

Because we are interested in the low-energy physics near the Dirac points $(0, 0, \pm k_0)$, symmetry-lowering lattice distortion terms can be classified order by order with respect to the momentum. Then, the leading terms are $1, k_z$ among $1, k_x, k_y, k_z$. Due to $TP$ symmetry, the $1$ can be combined with $\Gamma_0$ and $\Gamma_5$, and these terms are included in the $A_{1g}$ class. Because $A_{1g}$ class does not break any symmetry, we can ignore these terms. On the other hand, $k_z$ can be combined with $\Gamma_1$, $\Gamma_2$, $\Gamma_3$, and $\Gamma_4$. In the leading order, these four types of symmetry-lowering lattice distortions are dominant terms as discussed in the main text. Up to the quadratic order of momenta, the possible lattice distortion terms are listed in Table S3.

### S2.3. Lattice Hamiltonian under lattice distortions

For definiteness, we consider the explicit perturbation Hamiltonian in the lattice model. Then, we will show that how the lattice distortion terms generate the Dirac mass and shift the Dirac points in the low-energy effective Hamiltonian.

First, consider the $n_1$ and $n_2$ type lattice distortions. In this case, the coefficient functions $d_i(\mathbf{k})$ are given by

$$\begin{aligned} d_1(\mathbf{k}) &= n_{1,1}\sin k_x + n_{2,1}\sin k_y, \\ d_2(\mathbf{k}) &= -n_{1,1}\sin k_y + n_{2,1}\sin k_y, \\ d_3(\mathbf{k}) &= n_{1,0}\sin k_z, \\ d_4(\mathbf{k}) &= n_{2,0}\sin k_z, \\ d_5(\mathbf{k}) &= n_{1,2}(\cos k_x - \cos k_y) + n_{2,2}\sin k_x \sin k_y, \\ d_0(\mathbf{k}) &= n'_{1,2}(\cos k_x - \cos k_y) + n'_{2,2}\sin k_x \sin k_y, \end{aligned}$$

where $n_{i,j}$ and $n'_{i,j}$ are the constant parameters that represent the strength of the $n_i$ type lattice distortions. The second subscript $j$ indicates the power of momentum near Dirac points $(0, 0, \pm k_0)$ according to Table S3. Near the Dirac point, $k_z$ is approximately constant while $k_x$ and $k_y$ are very small. This is the reason why we only consider the leading terms $d_3(\mathbf{k}) = n_1 \sin k_z$ and $d_4(\mathbf{k}) = n_2 \sin k_z$ for $n_1$ and $n_2$ type lattice distortions in the main text. If we set $n_1 \equiv n_{1,0}$ and $n_2 \equiv n_{2,0}$, then near the Dirac points $(0, 0, \pm k_0)$ the low energy effective Hamiltonian is given by

$$H^{(\pm)}_{\text{Dirac}} = vk_x\Gamma_1 + vk_y\Gamma_2 \pm v_z(k_z \mp k_0)\Gamma_5 \pm n_1 \sin k_0 \Gamma_3 \pm n_2 \sin k_0 \Gamma_4. \tag{5}$$

Thus, the $n_1$ and $n_2$ type lattice distortions generate the Dirac mass terms.

Next, consider the $n_3$ type lattice distortion. Then, the coefficient functions are given by

$$\begin{aligned} d_1(\mathbf{k}) &= 0, \\ d_2(\mathbf{k}) &= n_{3,0}\sin k_z, \\ d_3(\mathbf{k}) &= n_{3,1}\sin k_y, \\ d_4(\mathbf{k}) &= 0, \\ d_5(\mathbf{k}) &= n_{3,2}\sin k_y \sin k_z, \\ d_0(\mathbf{k}) &= n'_{3,2}\sin k_y \sin k_z. \end{aligned}$$

Similar to the $n_1$ and $n_2$ case, the $n_{3,0}\sin k_z$ term is dominant near the Dirac point. In this case, however, the gap minimum points are shifted along $k_y$ axis from Dirac points $(0,0,\pm k_0)$ to $(0,\pm k_y^{(0)},\pm k_0)$, where $k_y^{(0)} = -n_{3,0}\sin k_0/v$ in the linear order. Then, the low energy effective Hamiltonian is given by

$$H^{(\pm)}_{\text{Dirac}} = vk_x\Gamma_1 + v(k_y \mp k_y^{(0)})\Gamma_2 \pm v_z(k_z \mp k_0)\Gamma_5 \pm m\Gamma_3, \tag{6}$$

where

$$m = -(\beta+\gamma)\frac{\sin^3 k_0 n_{3,0}^2}{2v^2} + n_{3,1}k_y^{(0)}. \tag{7}$$

Finally, consider the $n_4$ type lattice distortion, where the coefficient functions are given by

$$\begin{aligned}
d_1(\mathbf{k}) &= n_{4,0}\sin k_z,\\
d_2(\mathbf{k}) &= 0,\\
d_3(\mathbf{k}) &= n_{4,1}\sin k_x,\\
d_4(\mathbf{k}) &= 0,\\
d_5(\mathbf{k}) &= n_{4,2}\sin k_x \sin k_z,\\
d_0(\mathbf{k}) &= n'_{4,2}\sin k_x \sin k_z.
\end{aligned}$$

Similar to $n_3$ case, the Dirac points are shifted along $k_x$ axis from $(0,0,\pm k_0)$ to $(\pm k_y^{(0)},0,\pm k_0)$, where $k_z^{(0)} = -n_{4,0}\sin k_0/v$ in the linear order. The low-energy effective Hamiltonian is given by

$$H^{(\pm)}_{\text{Dirac}} = v(k_x \mp k_x^{(0)})\Gamma_1 + vk_y\Gamma_2 \pm v_z(k_z \mp k_0)\Gamma_5 \pm m\Gamma_3, \tag{8}$$

where

$$m = -(\beta+\gamma)\frac{\sin^3 k_0 n_{4,0}^2}{2v^2} + n_{4,1}k_x^{(0)}. \tag{9}$$

### S2.4. Enhancement of DOS at Fermi surface

In this subsection, we analytically calculate the DOS at Fermi surface using the low energy effective Dirac Hamiltonian. First, consider the $n_1$ and $n_2$ type lattice distortions. Because the energy eigenvalue is given by

$$E = \pm\sqrt{v^2(k_x^2+k_y^2) + v_z^2 q_z^2 + |n|^2\sin^2 k_0}, \quad |n| = \sqrt{n_1^2+n_2^2}, \tag{10}$$

the Fermi surface is given by the surface of an ellipsoid:

$$\left[\frac{k_x}{1/v}\right]^2 + \left[\frac{k_y}{1/v}\right]^2 + \left[\frac{k_z\mp k_0}{1/v_z}\right]^2 = E^2 - |n|^2\sin^2 k_0. \tag{11}$$

Since the ellipsoid $(x/a)^2+(y/b)^2+(z/c)^2 = 1$ has the volume $(4\pi/3)abc$, the electron number density $\mathcal{N}$ up to energy $E$ is given by

$$\mathcal{N}(E) = \frac{1}{3\pi v^2 v_z}[E^2 - |n|^2\sin^2 k_0]^{3/2}. \tag{12}$$

In the absence of lattice distortion ($|n| = 0$), the initial electron number density with the initial chemical potential $\mu_0$ is given by $\mathcal{N}(\mu_0) = \frac{1}{3\pi v^2 v_z}[\mu_0^2]^{3/2}$. In the presence of lattice distortion, $\mathcal{N}(\mu) = \frac{1}{3\pi v^2 v_z}[\mu(n)^2 - |n|^2\sin^2 k_0]^{3/2}$. The electron number density conservation gives

$$\mu(|n|) = \sqrt{\mu_0^2 + |n|^2\sin^2 k_0}.$$

Therefore, the Fermi surface for chemical potential $\mu(n)$ is given by

$$\left[\frac{k_x}{1/v}\right]^2 + \left[\frac{k_y}{1/v}\right]^2 + \left[\frac{k_z\mp k_0}{1/v_z}\right]^2 = \mu_0^2, \tag{13}$$

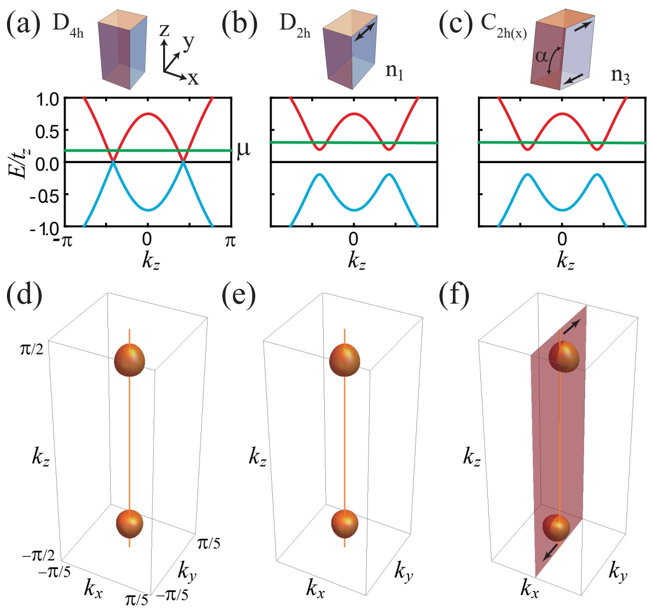


Fig. S1. **Band structures and Fermi surfaces for low-energy effective Dirac Hamiltonians. (a-c)** (a), (b), and (c) are band structures for the tetragonal, orthorhombic, and monoclinic crystal systems, respectively. The green lines indicate the chemical potentials. In the insets of (b) and (c), black arrows express the corresponding lattice distortions. Hence, in (b) [(c)], $n_1$ type ($n_3$ type) lattice distortion is turned on. **(d-f)** (d), (e), and (f) are Fermi surfaces for each crystal system. For the low-energy effective theory, the shape of the Fermi surfaces in (d), (e), and (f) are the same, but the position of the Fermi surface in (f) is shifted along the $k_y$ direction.

where the lattice distortions terms in the right-hand side are canceled. From Eq. (12), DOS for an arbitrary energy level $E$ is given by:

$$\mathrm{DOS}(E) = \frac{1}{\pi v^2 v_z} E[E^2 - |n|^2 \sin^2 k_0]^{1/2}. \tag{14}$$

Using the chemical potential $\mu(n)$, the lattice-distortion dependent DOS is given by

$$\mathrm{DOS}(\mu(|n|)) = \frac{1}{\pi v^2 v_z} \mu_0 [\mu_0^2 + |n|^2 \sin^2 k_0]^{1/2}. \tag{15}$$

Therefore, the DOS is enhanced by the lattice distortions.

For $n_3$ type lattice distortion, one can apply the above calculation simply by substitute the $|n|^2 \sin^2 k_0$ by $m^2$ using Eq. (7). The only difference is the $y$ directional shift of Fermi surface. The result is as follows: The chemical potential is given by

$$\mu(n_3) = \sqrt{\mu_0^2 + m^2}, \tag{16}$$

where $\mu_0$ is the chemical potential in the absence of the lattice distortion. The lattice-distortion dependent DOS is given by

$$\mathrm{DOS}(\mu(n_3)) = \frac{1}{\pi v^2 v_z} \mu_0 [\mu_0^2 + m^2]^{1/2}. \tag{17}$$

The Fermi surface for the chemical potential $\mu(n_3)$ is given by

$$\left[\frac{k_x}{1/v}\right]^2 + \left[\frac{k_y \mp k_y^{(0)}}{1/v}\right]^2 + \left[\frac{k_z \mp k_0}{1/v_z}\right]^2 = \mu_0^2. \tag{18}$$

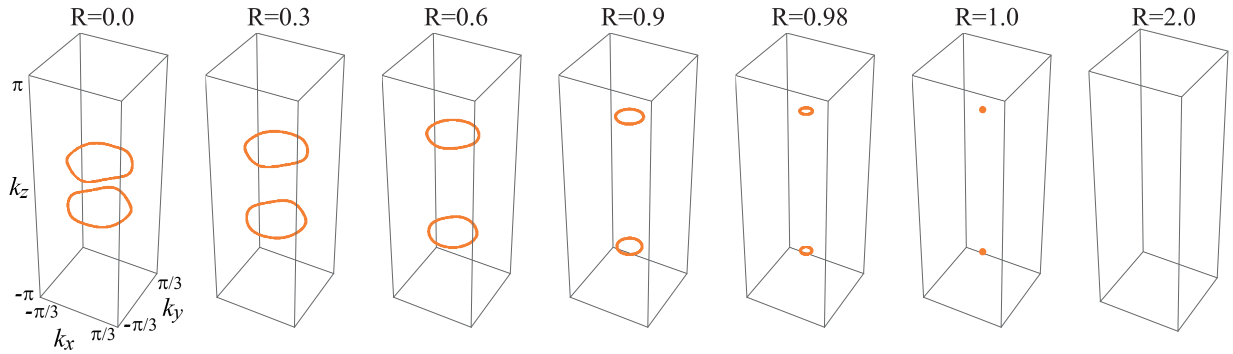


Fig. S2. **Evolution of nodal structures from $\Delta_1'$ to $\Delta_1$ phases.** $R$ is the evolution parameter which is given by $R = \Delta_1/\Delta_1'$. $R = 0$ and $R = 1$ indicate $\Delta_1'$ and $\Delta_1$ phases, respectively. As $R$ increases, two nodal rings becomes two point nodes on the $k_z$-axis when $\Delta_1 = \Delta_1'$. In this figure, the parameters are $M = 4.25t_z$, $t_{xy} = 2.0t_z$, $v = t_z$, $\beta = \gamma = 0$, $\Delta_1' = 0.05t_z$ and $\mu = 0.7t_z$. All parameters are scales in the unit of $t_z$.

## S3. SUPERCONDUCTING GAP STRUCTURE

### S3.1. Under $n_1$ and $n_2$ type lattice distortions

In this subsection, we discuss the superconducting gap structures for each phases under $n_1$ and $n_2$ type lattice distortions.

#### *S3.1.1. $\Delta_1$ and $\Delta_1'$ phases*

First, we consider the superconducting phases with $\Delta_1$ and $\Delta_1'$ paring potentials. Because they are included in the same representation regardless of lattice distortions, the mixture of $\Delta_1$ and $\Delta_1'$ phases is generally allowed. In this case, the energy eigenvalue $\mathcal{E}(\mathbf{k})$ is given by

$$\mathcal{E}(\mathbf{k}) = \pm\sqrt{|a|^2 + \mu^2 + \langle\Delta_1\rangle^2 + \langle\Delta_1'\rangle^2 \pm 2\sqrt{\mu^2 |a|^2 + \langle\Delta_1'\rangle^2 \left(|a|^2 - a_5^2\right) + \langle\Delta_1\rangle^2 \langle\Delta_1'\rangle^2 + 2\mu a_5 \left|\langle\Delta_1\rangle \langle\Delta_1'\rangle\right|}},$$

where $|a| = \sqrt{\sum_{i=1}^{5} a_i(\mathbf{k})^2}$. By solving $\mathcal{E}(\mathbf{k}) = 0$, the equations for nodal points are obtained:

$$|a|^2 = \mu^2 - \langle\Delta_1\rangle^2 + \langle\Delta_1'\rangle^2\,, \tag{19}$$

$$a_5(\mathbf{k})\langle\Delta_1'\rangle - \mu\langle\Delta_1\rangle = 0. \tag{20}$$

In general, for $\langle\Delta_1\rangle \neq 0$ and $\langle\Delta_1'\rangle \neq 0$, the number of momentum variable ($N_V = 3$) is larger than the number of equation ($N_E = 2$) and hence the one-dimensional solution is generally allowed. However, if $\langle\Delta_1\rangle > \langle\Delta_1'\rangle$, Eq. (20) can not be satisfied, which leads to a gapped phase. Therefore, the phase transition between full gap and nodal line phases occurs as shown in Fig. S2.

#### *S3.1.2. $\Delta_2$ and $\Delta_3$ phases*

Next, we consider the $\Delta_2$ and $\Delta_3$ superconducting phases. As discussed in the main text, these two paring potentials can be included in the same or different classes depending on the unbroken point group. In general, the energy eigenvalue is given by

$$\mathcal{E}(\mathbf{k}) = \pm\sqrt{|a|^2 + \mu^2 + \langle\Delta_2\rangle^2 + \langle\Delta_3\rangle^2 \pm 2\sqrt{\mu^2 |a|^2 + a_5^2(\langle\Delta_2\rangle^2 + \langle\Delta_3\rangle^2) + |a_4\langle\Delta_2\rangle + a_3\langle\Delta_3\rangle|^2}}.$$

By solving $\mathcal{E}(\mathbf{k}) = 0$, the following equations for nodal points are obtained:

$$|a|^2 = \mu^2 + \langle\Delta_2\rangle^2 + \langle\Delta_3\rangle^2\,, \quad a_1(\mathbf{k}) = a_2(\mathbf{k}) = a_3(\mathbf{k})\langle\Delta_2\rangle - a_4(\mathbf{k})\langle\Delta_3\rangle = 0. \tag{21}$$

These equations do not seem to allow any nodal point because the number of equation ($N_E = 4$) is larger than the number of momenta ($N_V = 3$). However, if there are mirror symmetries, then the existence of nodal points are guaranteed. Let us consider following cases:

(i) For $D_{4h}$ case, $C_{4z}$ symmetry protects the nodal points on the $k_z$ axis. The $C_{4z}$ symmetry restricts that $a_1(\mathbf{k}) = a_2(\mathbf{k}) = a_3(\mathbf{k}) = a_4(\mathbf{k}) = 0$ on the $k_z$ axis. Then, Eq. (21) on the $k_z$ axis becomes

$$(a_5(\mathbf{k}))^2 = \mu^2 + \langle\Delta_2\rangle^2 + \langle\Delta_3\rangle^2 .$$

On the $k_z$ axis, the number of variables ($N_V = 1$) is equal to the number of equations ($N_E = 1$). Thus, for $D_{4h}$ case, each $\Delta_2$ and $\Delta_3$ phases have nodal points on the $k_z$ axis.

(ii) For $D_{2h}$ case, $M_{yz}$ and $M_{xz}$ symmetries protect the nodal points on the $k_z$ axis. $a_1(\mathbf{k})$ and $a_4(\mathbf{k})$ are odd-functions under $M_{yz}$ operation, which leads that $a_1(\mathbf{k}) = a_4(\mathbf{k}) = 0$ on the corresponding mirror plane $(0, k_y, k_z)$. Similarly, $M_{xz}$ symmetry gives $a_2(\mathbf{k}) = a_4(\mathbf{k}) = 0$ on the corresponding mirror plane $(k_x, 0, k_z)$. Thus, both $M_{yz}$ and $M_{xz}$ symmetries endow $a_1(\mathbf{k}) = a_2(\mathbf{k}) = a_4(\mathbf{k}) = 0$ on the $k_z$ axis. Then, Eq. (21) on the $k_z$ axis becomes

$$|a|^2 = \mu^2 + \langle\Delta_2\rangle^2 + \langle\Delta_3\rangle^2 , \quad a_3(\mathbf{k})\langle\Delta_2\rangle = 0.$$

Since $a_3(\mathbf{k})$ is generally not zero, there can exist a nodal point only when $\langle\Delta_2\rangle = 0$. Thus, the $\Delta_3$ phase can have nodal points in $D_{2h}$ case.

(iii) For $D'_{2h}$ case, $M_{(110)}$ and $M_{(1\bar{1}0)}$ symmetries protect the nodal points on the $k_z$ axis. Using the transformation properties in Table 2 in the main text, we get

$$\begin{aligned} a_1(-k_y, -k_x, k_z) &= -a_2(k_x, k_y, k_z), \\ a_2(-k_y, -k_x, k_z) &= -a_1(k_x, k_y, k_z), \\ a_3(-k_y, -k_x, k_z) &= -a_3(k_x, k_y, k_z), \end{aligned}$$

under $M(110)$ operation, which leads that $a_1(\mathbf{k}) = -a_2(\mathbf{k})$ and $a_3(\mathbf{k}) = 0$ on the $k_z$ axis. Similarly, $M(1\bar{1}0)$ operation gives that $a_1(\mathbf{k}) = a_2(\mathbf{k})$ on the $k_z$ axis. Combining the results of two mirror symmetries, we get $a_1(\mathbf{k}) = a_2(\mathbf{k}) = a_3(\mathbf{k}) = 0$ on the $k_z$ axis. Then, Eq. (21) on the $k_z$ axis becomes

$$|a|^2 = \mu^2 + \langle\Delta_2\rangle^2 + \langle\Delta_3\rangle^2 , \quad a_4(\mathbf{k})\langle\Delta_3\rangle = 0.$$

Since $a_4(\mathbf{k})$ is generally not zero, there can exist a solution for these equations only when $\Delta_3 = 0$. Thus, the $\Delta_2$ phase can have nodal points in $D_{2h}$ case.

(iv) For $C_{2h(z)}$ case, there is no symmetry that protects nodal points.

### *S3.1.3.* $\Delta_{41}$ *and* $\Delta_{42}$ *phases*

Let us consider $\Delta_{41}$ and $\Delta_{42}$ phases. Like the previous cases, two paring potentials can be included in the same or different classes depending on the unbroken point group. The energy eigenvalue is given by

$$\mathcal{E}(\mathbf{k}) = \pm\sqrt{|a|^2 + \mu^2 + \langle\Delta_{41}\rangle^2 + \langle\Delta_{42}\rangle^2 \pm 2\sqrt{\mu^2|a|^2 + |a_2\langle\Delta_{41}\rangle - a_1\langle\Delta_{42}\rangle|^2 + a_5^2\left(\langle\Delta_{41}\rangle^2 + \langle\Delta_{42}\rangle^2\right)}},$$

By solving $\mathcal{E}(\mathbf{k}) = 0$, the following set of equations for nodal points is obtained:

$$|a|^2 = \mu^2 + \langle\Delta_{41}\rangle^2 + \langle\Delta_{42}\rangle^2 , \quad a_3(\mathbf{k}) = a_4(\mathbf{k}) = \langle\Delta_{41}\rangle a_1(\mathbf{k}) + \langle\Delta_{42}\rangle a_2(\mathbf{k}) = 0. \tag{22}$$

These equations do not seem to allow any nodal point because the number of equation ($N_E = 4$) is larger than the number of momenta ($N_V = 3$). However, mirror symmetries can guarantee nodal points. Let us consider the following cases:

(i) For $D_{4h}$ case, $C_{4z}$ symmetry seems to allow the existence of the nodal points on the $k_z$ axis. The $C_{4z}$ symmetry restricts that $a_1(\mathbf{k}) = a_2(\mathbf{k}) = a_3(\mathbf{k}) = a_4(\mathbf{k}) = 0$ on the $k_z$ axis. Then, Eq. (22) on the $k_z$ axis become

$$a_5(\mathbf{k})^2 = \mu^2 + |\Delta_{41}|^2 + |\Delta_{42}|^2 .$$

On the $k_z$ axis, the number of variables ($N_V = 1$) is equal to the number of equations ($N_E = 1$), which seems to allow nodal points. However, these nodal points are accidental nodal points because the $D_{4h}$ symmetry is spontaneously broken when $\Delta_{41}$ or $\Delta_{42}$ pairing potentials are present.

(ii) For $D_{2h}$ case, $M_{yz}$ and $M_{xz}$ symmetries protect the nodal points on mirror plane. $a_1(\mathbf{k})$ and $a_4(\mathbf{k})$ are odd-functions under $M_{yz}$ operation, which leads to $a_1(\mathbf{k}) = a_4(\mathbf{k}) = 0$ on the corresponding mirror plane $(0, k_y, k_z)$. Thus, for $\Delta_{41}$ phase, Eq. (22) becomes

$$|a|^2 = \mu^2 + \langle\Delta_{41}\rangle^2 , \quad a_3(\mathbf{k}) = 0.$$

Because the number of equation ($N_E = 2$) is equal to the number of momenta ($N_V = 2$) on the mirror plane, which can allow nodal points. Similarly, $M_{xz}$ symmetry gives $a_2(\mathbf{k}) = a_4(\mathbf{k}) = 0$ on the corresponding mirror plane $(k_x, 0, k_z)$. Thus, for $\Delta_{42}$ phase, Eq. (22) becomes

$$|a|^2 = \mu^2 + \langle\Delta_{42}\rangle^2, \quad a_3(\mathbf{k}) = 0.$$

Hence, $\Delta_{41}$ and $\Delta_{42}$ phases can have nodal points in the mirror planes.

(iii) For $D'_{2h}$ case, $M_{(110)}$ and $M_{(1\bar{1}0)}$ symmetries allow the nodal points on the corresponding mirror plane. Because there are diagonal mirror symmetries, the following linear combinations of the pairing potentials are meaningful and do not break the $D'_{2h}$ point group symmetry.

$$\Delta_+ = \Delta_{41} + \Delta_{42}, \quad \Delta_- = \Delta_{41} - \Delta_{42}.$$

Under $M_{(110)}$ operation,

$$a_1(-k_y, -k_x, k_z) = -a_2(k_x, k_y, k_z),$$
$$a_2(-k_y, -k_x, k_z) = -a_1(k_x, k_y, k_z),$$
$$a_3(-k_y, -k_x, k_z) = -a_3(k_x, k_y, k_z),$$

which leads to $a_1(\mathbf{k}) = -a_2(\mathbf{k})$ and $a_3(\mathbf{k}) = 0$ on the $M_{(110)}$ mirror plane. Thus, for $\Delta_+$ phase with the condition $\langle\Delta_-\rangle = 0$, Eq. (22) becomes

$$|a|^2 = \mu^2 + 2\langle\Delta_+\rangle^2, \quad a_4(\mathbf{k}) = 0.$$

Because the number of equation ($N_E = 2$) is equal to the number of momenta ($N_V = 2$) on the mirror plane, which can allow nodal points. Similarly, $M_{(1\bar{1}0)}$ operation gives that $a_1(\mathbf{k}) = a_2(\mathbf{k})$ on the $k_z$ axis. Thus, for $\Delta_-$ phase with the condition $\langle\Delta_+\rangle = 0$, Eq. (22) becomes

$$|a|^2 = \mu^2 + 2\langle\Delta_-\rangle^2, \quad a_4(\mathbf{k}) = 0.$$

Hence, $\Delta_+$ and $\Delta_-$ phases can have nodal points in the mirror planes.

(iv) For $C_{2h(z)}$ case, there is no symmetry that allows a nodal point.

### S3.2. Under $n_3$ type lattice distortions

So far, we have discussed the nodal structure of superconducting phases for the point groups $D_{4h}$, $D_{2h}$, $D'_{2h}$, and $C_{2h(z)}$ under $n_1$ and $n_3$ type lattice distortions. Now, we discuss $C_{2h(x)}$ point group under $n_3$ type lattice distortion. In this case, the classification of pairing potentials are much different from the previous case: $\Delta_2$ and $\Delta_{42}$ are included in $A_u$ representation. $\Delta_3$ and $\Delta_{41}$ are included in $B_u$ representation. Therefore, mixed phases are generally allowed. The mixed phase composed of $\Delta_2$ and $\Delta_{42}$ is fully gapped [Fig. 2(e) in the main text]. On the other hand, there are nodal points in mixed phase composed of $\Delta_3$ and $\Delta_{41}$ pairing potentials [Fig. 2(e) in the main text]. The existence of such nodal points can be shown using the $M_{yz}$ mirror symmetry which is not broken in the $C_{2h(x)}$ point group.

(1) For $\Delta_2$ and $\Delta_{42}$ phases, the energy eigenvalue is given by

$$\mathcal{E}(\mathbf{k}) = \pm\sqrt{|a|^2 + \mu^2 + \langle\Delta_2\rangle^2 + \langle\Delta_{42}\rangle^2 \pm 2\sqrt{\mu^2 |a|^2 + |a_4\langle\Delta_2\rangle + a_1\langle\Delta_{42}\rangle|^2 + a_5^2\left(\langle\Delta_2\rangle^2 + \langle\Delta_{42}\rangle^2\right)}},$$

The nodal equations are given by

$$|a|^2 = \mu^2 + \langle\Delta_2\rangle^2 + \langle\Delta_{42}\rangle^2, \quad a_2(\mathbf{k}) = a_3(\mathbf{k}) = \langle\Delta_2\rangle a_1(\mathbf{k}) - \langle\Delta_{42}\rangle a_4(\mathbf{k}) = 0.$$

Because the number of equations ($N_E = 4$) is larger than the number of variables ($N_V = 3$), there is no nodal point.

(2) For $\Delta_3$ and $\Delta_{41}$ phases, the energy eigenvalue is given by

$$\mathcal{E}(\mathbf{k}) = \pm\sqrt{|a|^2 + \mu^2 + \langle\Delta_3\rangle^2 + \langle\Delta_{41}\rangle^2 \pm 2\sqrt{\mu^2 |a|^2 + |a_3\langle\Delta_3\rangle - a_2\langle\Delta_{41}\rangle|^2 + a_5^2\left(\langle\Delta_3\rangle^2 + \langle\Delta_{41}\rangle^2\right)}},$$

The nodal equations are given by

$$|a|^2 = \mu^2 + \langle\Delta_3\rangle^2 + \langle\Delta_{41}\rangle^2, \quad a_1(\mathbf{k}) = a_4(\mathbf{k}) = \langle\Delta_3\rangle a_2(\mathbf{k}) - \langle\Delta_{41}\rangle a_3(\mathbf{k}) = 0.$$

The $M_{yz}$ symmetry gives $a_1(\mathbf{k}) = a_4(\mathbf{k}) = 0$ in the mirror plane. Then, the nodal equations in the mirror plane are given by

$$|a|^2 = \mu^2 + \langle\Delta_3\rangle^2 + \langle\Delta_{41}\rangle^2, \quad \langle\Delta_3\rangle a_2(\mathbf{k}) - \langle\Delta_{41}\rangle a_3(\mathbf{k}) = 0.$$

Because the number of equation ($N_E = 2$) is equal to the number of momenta ($N_V = 2$) on the mirror plane, nodal points are allowed.

## S4. STABILITY OF NODAL STRUCTURES

### S4.1. Chiral winding number

We show that the chiral winding number for inversion-even-parity (inversion-odd-parity) superconductor is nontrivial (trivial). Because the BdG Hamiltonian has a chiral symmetry $\{\Gamma, H_{\mathrm{BdG}}(\mathbf{k})\} = 0$, one can define a chiral winding number for a circle enclosing a Dirac point in the Brillouin zone [11–13].

$$W = \frac{1}{4\pi i} \oint \mathrm{Tr}\left[\Gamma H_{\mathrm{BdG}}^{-1}(\mathbf{k}) dH_{\mathrm{BdG}}(\mathbf{k})\right]. \tag{23}$$

When the circle is parametrized by $\theta \in [0, 2\pi)$ via a function $\mathbf{k}(\theta)$, the winding number is given by

$$W = \frac{1}{4\pi i} \oint d\theta \mathrm{Tr}\left[\Gamma H_{\mathrm{BdG}}^{-1}(\mathbf{k}(\theta))\partial_\theta H_{\mathrm{BdG}}(\mathbf{k}(\theta))\right]. \tag{24}$$

Now we discuss the effect of $P$ and $T$ symmetries on the chiral winding number. Because the $PT$ symmetry gives a Kramer's doublet at each momentum in the BZ, the nodal points are four-fold degenerate. Since the two-fold degenerate gapless point play a role of a singularity source for the chiral winding number, a four-fold degenerate nodal point has two sources for the chiral winding number. Thus, the transformation properties of the chiral winding number under $P$ and $T$ transformation is crucial for non-vanishing winding number.

Under $P$ and $T$, the BdG Hamiltonian satisfies

$$(\tilde{P}T)H_{\mathrm{BdG}}(\mathbf{k})(\tilde{P}T)^{-1} = H_{\mathrm{BdG}}(\mathbf{k}), \tag{25}$$

$$(\tilde{P}T)H_{\mathrm{BdG}}^{-1}(\mathbf{k})(\tilde{P}T)^{-1} = H_{\mathrm{BdG}}^{-1}(\mathbf{k}). \tag{26}$$

Let

$$\Gamma(\tilde{P}T) = \eta_{\Gamma,\tilde{P}T}(\tilde{P}T)\Gamma, \tag{27}$$

where $\eta_{\Gamma,\tilde{P}T} = \pm 1$. For inversion-even and inversion-odd parity superconductors, the values of $\eta_{\Gamma,\tilde{P}T}$ are $-1$ and $+1$, respectively. The transformation property of the winding number under $PT$ transformation is given by

$$\begin{aligned}
W &= \frac{1}{4\pi i} \oint d\theta \mathrm{Tr}\left[\Gamma H_{\mathrm{BdG}}^{-1}(\mathbf{k}(\theta))\partial_\theta H_{\mathrm{BdG}}(\mathbf{k}(\theta))\right], && (28)\\
&= \frac{1}{4\pi i} \oint d\theta \mathrm{Tr}\left[\Gamma(\tilde{P}T)H_{\mathrm{BdG}}^{-1}(\mathbf{k}(\theta))(\tilde{P}T)^{-1}\partial_\theta(\tilde{P}T)H_{\mathrm{BdG}}(\mathbf{k}(\theta))(\tilde{P}T)^{-1}\right], && (29)\\
&= \frac{1}{4\pi i} \oint d\theta \mathrm{Tr}\left[\Gamma(\tilde{P}is_yK)H_{\mathrm{BdG}}^{-1}(\mathbf{k}(\theta))\tilde{P}(-is_yK)\partial_\theta(\tilde{P}is_yK)H_{\mathrm{BdG}}(\mathbf{k}(\theta))\tilde{P}(-is_yK)\right], && (30)\\
&= \frac{1}{4\pi i} \oint d\theta \mathrm{Tr}\left[\tilde{P}(-is_y)\Gamma\tilde{P}(is_y)(H_{\mathrm{BdG}}^{-1}(\mathbf{k}(\theta)))^*\partial_\theta H_{\mathrm{BdG}}(\mathbf{k}(\theta))^*\right], && (31)\\
&= \frac{1}{4\pi i} \oint d\theta \mathrm{Tr}\left[(\tilde{P}T)^{-1}\Gamma(\tilde{P}T)(H_{\mathrm{BdG}}^{-1}(\mathbf{k}(\theta)))\partial_\theta H_{\mathrm{BdG}}(\mathbf{k}(\theta))\right]^*, && (32)\\
&= \frac{\eta_{PT}^{\Gamma}}{4\pi i} \oint d\theta \mathrm{Tr}\left[\Gamma(H_{\mathrm{BdG}}^{-1}(\mathbf{k}(\theta)))\partial_\theta H_{\mathrm{BdG}}(\mathbf{k}(\theta))\right]^*, && (33)\\
&= \frac{\eta_{\Gamma,\tilde{P}T}}{4\pi i}\left[4\pi i W\right]^*, && (34)\\
&= -\eta_{\Gamma,\tilde{P}T} W. && (35)
\end{aligned}$$

Therefore, the chiral winding number for inversion-even (inversion-odd) parity superconductor is nontrivial (trivial).

### S4.2. Mirror chiral winding number

Let us investigate the mirror chiral winding number. Even though the chiral winding number is zero for the inversion-odd-parity superconductor, we can obtain a nontrivial mirror chiral winding number using a mirror symmetry. The mirror chiral

winding number ($W_M$) can be defined for a circle $C$ which encloses the Dirac point in the mirror plane [14, 15]. When the circle $C$ is parametrized by $\theta \in [0, 2\pi)$, the mirror chiral winding number is given by

$$W_M = -\frac{1}{4\pi} \oint_C d\theta \mathrm{Tr} \left[ \tilde{M} \Gamma H_{\mathrm{BdG}}^{-1}(\mathbf{k}(\theta)) \partial_\theta H_{\mathrm{BdG}}(\mathbf{k}(\theta)) \right]. \tag{36}$$

First, we consider the transformation property of this winding number under chiral symmetry operation. Let

$$\Gamma \tilde{M} = \eta_{\tilde{M},\Gamma} \tilde{M} \Gamma, \tag{37}$$

where $\eta_{\tilde{M},\Gamma} = \pm 1$. The BdG Hamiltonian has the following chiral symmetry.

$$\Gamma H_{\mathrm{BdG}}(\mathbf{k}) \Gamma^{-1} = -H_{\mathrm{BdG}}(\mathbf{k}), \tag{38}$$

$$\Gamma H_{\mathrm{BdG}}^{-1}(\mathbf{k}) \Gamma^{-1} = -H_{\mathrm{BdG}}^{-1}(\mathbf{k}). \tag{39}$$

Then,

$$W_M = -\frac{1}{4\pi} \oint d\theta \mathrm{Tr} \left[ \tilde{M} \Gamma H_{\mathrm{BdG}}^{-1}(\mathbf{k}(\theta)) \partial_\theta H_{\mathrm{BdG}}(\mathbf{k}(\theta)) \right], \tag{40}$$

$$= -\frac{1}{4\pi} \oint d\theta \mathrm{Tr} \left[ \tilde{M} \Gamma \Gamma H_{\mathrm{BdG}}^{-1}(\mathbf{k}(\theta)) \Gamma^{-1} \partial_\theta \Gamma H_{\mathrm{BdG}}(\mathbf{k}(\theta)) \Gamma^{-1} \right], \tag{41}$$

$$= -\frac{1}{4\pi} \oint d\theta \mathrm{Tr} \left[ \Gamma^{-1} \tilde{M} \Gamma \Gamma H_{\mathrm{BdG}}^{-1}(\mathbf{k}(\theta)) \partial_\theta H_{\mathrm{BdG}}(\mathbf{k}(\theta)) \right], \tag{42}$$

$$= \eta_{\tilde{M},\Gamma} W_M. \tag{43}$$

This means that the mirror chiral winding number is nontrivial (zero) when the mirror operator and chiral operator commute (anticommute) with $\tilde{M}$. Because $\tilde{M} = M\tau_0$ ($\tilde{M} = M\tau_z$) for the mirror-even-parity (mirror-odd-parity) pairing potential, the mirror-even (mirror-odd) parity superconducting phase has nontrivial (zero) mirror chiral winding number.

Similarly, one can show that

$$W_M(C_{2z}\mathbf{k}) = \eta_{C_{2z}} W_M(\mathbf{k}), \tag{44}$$

where $\eta_{C_{2z}}$ is the parity of the pairing potential under $C_{2z}$ transformation.

### S4.3. Accidental point nodes in $\Delta_{41}$ and $\Delta_{42}$ phases

In the absence of lattice distortions, the nodal points on the $k_z$ axis in Fig. 2(a) are accidental nodal points. The nodal points are protected by two-fold rotation symmetry along $z$-axis ($\tilde{C}_{2z}$) and spin $z$-component conservation symmetry on the $k_z$ axis ($s_z$) if there exist the $D_{4h}$ point group symmetry. However, because the $D_{4h}$ symmetry is spontaneously broken when $\Delta_{41}$ or $\Delta_{42}$ pairing potentials are present, the nodal points are accidental nodal points.

The $\tilde{C}_{2z}$ operator for the BdG Hamiltonian $H_{\mathrm{BdG}}(\mathbf{k})$ is given by $\tilde{C}_{2z} = \mathrm{diag}[C_{2z}, \eta_{C_{2z}} s_y C_{2z}^* s_y] = -is_z\sigma_z\tau_z$, where $\eta_{C_{2z}} = -1$ is used for $\Delta_{41}$ and $\Delta_{42}$. The spin $z$-component conservation symmetry on the $k_z$ axis originates from the $C_{4z}$ symmetry of DSM. Because the DSM Hamiltonian $H(\mathbf{k})$ commutes with $C_{4z} = e^{-i(\pi/4)(2\sigma_0+\sigma_z)s_z}$ on the $k_z$ axis, its generator also commutes with the DSM Hamiltonian, which leads to $[H(k_z), s_z] = 0$ regardless of spin-orbit couplings. In addition, $\Delta_{41} = \sigma_x$ and $\Delta_{42} = \sigma_y s_z$ commute with $s_z$ operator. Thus, the BdG Hamiltonian also commutes with $s_z$ operator $[H_{\mathrm{BdG}}(k_z), s_z] = 0$, which means that the eigenvalue of $s_z$ operator is a good quantum numbers on the $k_z$ axis. Due to these symmetries, $H_{\mathrm{BdG}}(k_z), \tilde{C}_{2z}$, and $s_z$ commute on the $k_z$ axis and the energy bands can be labeled by the eigenvalues of the $\tilde{C}_{2z}$ and $s_z$ operators. Because four wavefunctions at the same momentum, $|\psi\rangle, PT|\psi\rangle, PC|\psi\rangle, TC|\psi\rangle$, have different eigenvalues, which allows an accidental nodal point.

The details are as follows. The $\tilde{C}_{2z}$ operator satisfies the following commutation relations:

$$\tilde{C}_{2z}\tilde{P}T = \tilde{P}T\tilde{C}_{2z}, \tag{45}$$

$$\tilde{C}_{2z}\tilde{P}C = -\tilde{P}C\tilde{C}_{2z}. \tag{46}$$

So, if a wavefunction $|\Psi_{\mathbf{k}}\rangle$ at momentum $\mathbf{k}$ has an eigenvalue $\lambda = \pm i$ of $\tilde{C}_{2z}$, the $PT$ and $PC$ transformed wave functions satisfy the follow eigenvalue equations:

$$\tilde{C}_{2z}(\tilde{P}T|\Psi_{\mathbf{k}}\rangle) = -\lambda(\tilde{P}T|\Psi_{\mathbf{k}}\rangle), \tag{47}$$

$$\tilde{C}_{2z}(\tilde{P}C|\Psi_{\mathbf{k}}\rangle) = +\lambda(\tilde{P}C|\Psi_{\mathbf{k}}\rangle), \tag{48}$$

where $\tilde{P}T = (\sigma_z\tau_z)(is_yK)$ and $\tilde{P}C = (\sigma_z\tau_z)(is_y\tau_yK)$. Similarly, for $s_z$,

$$s_z\tilde{P}T = -\tilde{P}Ts_z, \tag{49}$$

$$s_z\tilde{P}C = -\tilde{P}Cs_z, \tag{50}$$

and if $s_z\left|\Psi_{\mathbf{k}}\right\rangle = s\left|\Psi_{\mathbf{k}}\right\rangle$, then

$$s_z(\tilde{P}T\left|\Psi_{\mathbf{k}}\right\rangle) = -s(\tilde{P}T\left|\Psi_{\mathbf{k}}\right\rangle), \tag{51}$$

$$s_z(\tilde{P}C\left|\Psi_{\mathbf{k}}\right\rangle) = -s(\tilde{P}C\left|\Psi_{\mathbf{k}}\right\rangle). \tag{52}$$

Thus, four energy bands at the nodal points can be labeled by the different eigenvalues of the $\tilde{C}_{2z}$ and $s_z$ operators:

$$\left|\Psi_{\mathbf{k}}\right\rangle = \left|+s,+\lambda\right\rangle, \quad \tilde{P}T\left|\Psi_{\mathbf{k}}\right\rangle = \left|-s,-\lambda\right\rangle, \quad \tilde{P}C\left|\Psi_{\mathbf{k}}\right\rangle = \left|-s,+\lambda\right\rangle, \quad TC\left|\Psi_{\mathbf{k}}\right\rangle = \left|+s,-\lambda\right\rangle, \tag{53}$$

which protects the nodal points on the $k_z$ axis.

Note that the existence of the accidental point nodes also can be verified via $C_{4z}$ symmetry [15, 16].

## S5. SUPERCONDUCTING GAP EQUATIONS

This section calculates the superconducting critical temperature for various pairing potentials in the weak coupling limit by solving linearized gap equations [15, 17–20]. The linearized gap equations for all superconducting phases are given by

$$\begin{vmatrix} U\chi_1(T_c)-1 & U\chi_{1,1'}(T_c) \\ U\chi_{1,1'}(T_c) & U\chi_{1'}(T_c)-1 \end{vmatrix} = 0, \quad \text{for } \Delta_1 \text{ and } \Delta_1' \text{ phases}, \tag{54}$$

$$\begin{vmatrix} V\chi_2(T_c)-1 & V\chi_{2,3}(T_c) \\ V\chi_{2,3}(T_c) & V\chi_3(T_c)-1 \end{vmatrix} = 0, \quad \text{for } \Delta_2 \text{ and } \Delta_3 \text{ phases}, \tag{55}$$

$$\begin{vmatrix} V\chi_{41}(T_c)-1 & V\chi_{41,42}(T_c) \\ V\chi_{41,42}(T_c) & V\chi_{42}(T_c)-1 \end{vmatrix} = 0, \quad \text{for } \Delta_{41} \text{ and } \Delta_{41} \text{ phases}, \tag{56}$$

$$\begin{vmatrix} V\chi_2(T_c)-1 & V\chi_{2,42}(T_c) \\ V\chi_{2,42}(T_c) & V\chi_{42}(T_c)-1 \end{vmatrix} = 0, \quad \text{for } \Delta_2 \text{ and } \Delta_{42} \text{ phases}, \tag{57}$$

$$\begin{vmatrix} V\chi_3(T_c)-1 & V\chi_{3,41}(T_c) \\ V\chi_{3,41}(T_c) & V\chi_{41}(T_c)-1 \end{vmatrix} = 0, \quad \text{for } \Delta_3 \text{ and } \Delta_{41} \text{ phases}, \tag{58}$$

where the generalized susceptibility $\chi_i$ is given by

$$\chi_i = \int \frac{d^3\mathbf{k}}{(2\pi)^3} f_i(\mathbf{k}) \frac{\tanh(\beta_c\varepsilon_{\mathbf{k}}/2)}{2\varepsilon_{\mathbf{k}}}, \tag{59}$$

where $f_i(\mathbf{k})$ is the form factor for each pairing potential. $\varepsilon_{\mathbf{k}} = |a| - \mu$ is the normal state energy measured from the Fermi level, where $|a| = \sqrt{\sum_{i=1}^{5} a_i(\mathbf{k})^2}$. For the off-diagonal terms in the gap equations, the the generalized susceptibilities $\chi_{i,j}$ are given by

$$\chi_{i,j} = \int \frac{d^3\mathbf{k}}{(2\pi)^3} f_{i,j}(\mathbf{k}) \frac{\tanh(\beta_c\varepsilon_{\mathbf{k}}/2)}{2\varepsilon_{\mathbf{k}}}. \tag{60}$$

The form factors for each pairing potentials are given by

$$f_1(\mathbf{k}) = 1, \quad f_{1'}(\mathbf{k}) = 1 - \frac{a_1(\mathbf{k})^2 + a_2(\mathbf{k})^2 + a_3(\mathbf{k})^2 + a_4(\mathbf{k})^2}{\sqrt{\mu^2\left|a\right|^2}}, \tag{61}$$

$$f_2(\mathbf{k}) = 1 - \frac{a_5(\mathbf{k})^2 + a_4(\mathbf{k})^2}{\sqrt{\mu^2\left|a\right|^2}}, \quad f_3(\mathbf{k}) = 1 - \frac{a_5(\mathbf{k})^2 + a_3(\mathbf{k})^2}{\sqrt{\mu^2\left|a\right|^2}}, \tag{62}$$

$$f_{41}(\mathbf{k}) = 1 - \frac{a_5(\mathbf{k})^2 + a_2(\mathbf{k})^2}{\sqrt{\mu^2\left|a\right|^2}}, \quad f_{42}(\mathbf{k}) = 1 - \frac{a_5(\mathbf{k})^2 + a_1(\mathbf{k})^2}{\sqrt{\mu^2\left|a\right|^2}}. \tag{63}$$

The form factors for the off-diagonal terms are given by

$$f_{1,1'}(\mathbf{k}) = -\frac{a_5(\mathbf{k})}{|a|},\ f_{2,3}(\mathbf{k}) = -\frac{a_3(\mathbf{k})a_4(\mathbf{k})}{\sqrt{\mu^2\,|a|^2}},\ f_{41,42}(\mathbf{k}) = \frac{a_1(\mathbf{k})a_2(\mathbf{k})}{\sqrt{\mu^2\,|a|^2}}, \tag{64}$$

$$f_{2,42}(\mathbf{k}) = -\frac{a_1(\mathbf{k})a_4(\mathbf{k})}{\sqrt{\mu^2\,|a|^2}},\ f_{3,41}(\mathbf{k}) = -\frac{a_2(\mathbf{k})a_3(\mathbf{k})}{\sqrt{\mu^2\,|a|^2}}. \tag{65}$$

Using the low-energy effective Hamiltonian near Dirac points, the gap equations can be further simplified. Under $n_1$, $n_2$, and $n_3$ type of lattice distortions, the energy eigenvalue of DSM near Dirac points $(0, \pm k_y^{(0)}, \pm k_0)$ is given by

$$E = \pm\sqrt{v^2k_x^2 + v^2(k_y \mp k_y^{(0)})^2 + v_z^2(k_z \mp k_0)^2 + \tilde{m}^2}, \tag{66}$$

where $k_y^{(0)} = -n_3 \sin k_0/v$, $\tilde{m}^2 = (n_1^2 + n_2^2)\sin^2 k_0 + m^2$, and $m = (\beta+\gamma)\frac{n_3^2 \sin^3 k_0}{2v^2}$. Then, the Fermi surface can be parameterized using the ellipsoidal coordinates.

$$k_x = \frac{r}{v}\sin\theta\cos\phi,\ k_y = \frac{r}{v}\sin\theta\sin\phi \pm k_y^{(0)},\ k_z = \frac{r}{v_z}\cos\theta \pm k_0. \tag{67}$$

Using this coordinates, the range of momentum integration in the susceptibility in Eqs. (59) and (60) is given by $\mu - \omega_D < \sqrt{r^2+\tilde{m}^2} < \mu + \omega_D$, $0 < \theta < \pi$, and $0 < \phi < 2\pi$ where $\omega_D$ is the effective thickness of the Fermi surface. In the weak coupling limit, the thickness of the Fermi surface is very shallow ($\omega_D \ll \mu$). Because there are two Fermi spheres, the factor 2 should be considered to calculate the susceptibilities. The Jacobian factor is given by

$$\text{Jacobian} = \left|\frac{r^2\sin\theta}{v^2 v_z}\right|. \tag{68}$$

Then, the momentum dependent form factor $f_i(\mathbf{k})$ in Eq. (59) become a function of $r, \theta$, and $\phi$, that is, $f_i(r,\theta,\phi)$. Therefore, using the ellipsoidal coordinates, the susceptibility can be approximated as direct product of two integration in the weak coupling limit ($\omega_D \ll \mu$).

$$\chi_i = 2\int_{\sqrt{(\mu-\omega_D)^2-\tilde{m}^2}}^{\sqrt{(\mu+\omega_D)^2-\tilde{m}^2}}\int_0^{\pi}\int_0^{2\pi}\frac{drd\theta d\phi}{(2\pi)^3}\left|\frac{r^2\sin\theta}{v^2v_z}\right| f_i(r,\theta,\phi)\frac{\tanh(\beta_c(\sqrt{r^2+\tilde{m}^2}-\mu)/2)}{(\sqrt{r^2+\tilde{m}^2}-\mu)} \tag{69}$$

$$= 2\int_{-\omega_D}^{+\omega_D}\int_0^{\pi}\int_0^{2\pi}\frac{(R+\mu)dR}{\sqrt{(R+\mu)^2-\tilde{m}^2}}\frac{d\theta d\phi}{(2\pi)^3}\left|\frac{[(R+\mu)^2-\tilde{m}^2]\sin\theta}{v^2v_z}\right| \times f_i(\sqrt{(R+\mu)^2-\tilde{m}^2},\theta,\phi)\frac{\tanh(\beta_c R/2)}{R}, \tag{70}$$

$$= 2\int_{-\omega_D}^{+\omega_D}\int_0^{\pi}\int_0^{2\pi}\frac{dRd\theta d\phi}{(2\pi)^3}\left|\frac{\mu\sqrt{\mu^2-\tilde{m}^2}\sin\theta}{v^2v_z}\right| f_i(\sqrt{\mu^2-\tilde{m}^2},\theta,\phi)\frac{\tanh(\beta_c R/2)}{R} + O\left(\frac{\omega_D}{\mu}\right)^2, \tag{71}$$

$$\approx \Omega(\mu, n_i)\ \mathcal{R}(\beta_c) \tag{72}$$

In the second line, the change of variable $R = \sqrt{r^2+\tilde{m}^2} - \mu$ is used. Therefore, the susceptibility is separated into the radial part and angular part. The radial part is given by

$$\mathcal{R}(\beta_c) = \int_{-\omega_D}^{\omega_D} dR\,\frac{\tanh(\beta_c R/2)}{R}, \tag{73}$$

where $R = |a| - \mu$ is the normal state energy measured from the Fermi level. The angular integral is given by

$$\Omega(\mu, n_i) = C_0\int_0^{\pi}\int_0^{2\pi} d\theta d\phi \sin\theta f_i(\sqrt{\mu^2-\tilde{m}^2},\theta,\phi). \tag{74}$$

where $C_0$ is the common coefficient $C_0 \equiv \frac{2}{(2\pi)^3}\frac{\mu\sqrt{\mu^2-\tilde{m}^2}}{v^2v_z}$. After the analytical integration, the following susceptibilities can be

obtained.

$$\chi_1 = 4\pi C_0 \mathcal{R}(T_c), \tag{75}$$

$$\chi_{1'} = \frac{4\pi}{3}\left(\frac{3\mu\sqrt{\mu^2-m^2}+2m^2-2\mu^2-n_1^2\sin^2 k_0-n_2^2\sin^2 k_0}{\mu\sqrt{\mu^2-m^2}}\right)C_0\mathcal{R}(T_c), \tag{76}$$

$$\chi_2 = \frac{4\pi}{3}\left(\frac{3\mu\sqrt{\mu^2-m^2}+m^2-\mu^2+n_1^2\sin^2 k_0-2n_2^2\sin^2 k_0}{\mu\sqrt{\mu^2-m^2}}\right)C_0\mathcal{R}(T_c), \tag{77}$$

$$\chi_3 = \frac{4\pi}{3}\left(\frac{3\mu\sqrt{\mu^2-m^2}+m^2-\mu^2-2n_1^2\sin^2 k_0+n_2^2\sin^2 k_0}{\mu\sqrt{\mu^2-m^2}}\right)C_0\mathcal{R}(T_c), \tag{78}$$

$$\chi_{41} = \frac{4\pi}{3}\left(\frac{3\mu\sqrt{\mu^2-m^2}+2\left(m^2+n_1^2\sin^2 k_0+n_2^2\sin^2 k_0\right)-2\mu^2}{\mu\sqrt{\mu^2-m^2}}\right)C_0\mathcal{R}(T_c), \tag{79}$$

$$\chi_{42} = \frac{4\pi}{3}\left(\frac{3\mu\sqrt{\mu^2-m^2}+2\left(m^2+n_1^2\sin^2 k_0+n_2^2\sin^2 k_0\right)-2\mu^2}{\mu\sqrt{\mu^2-m^2}}\right)C_0\mathcal{R}(T_c), \tag{80}$$

$$\chi_{2,3} = 4\pi\left(-\frac{n_1 n_2}{\mu\sqrt{\mu^2-m^2}}\right)C_0\mathcal{R}(T_c), \tag{81}$$

$$\chi_{1,1'} = \chi_{41,42} = \chi_{2,42} = \chi_{3,41} = 0. \tag{82}$$

Using these susceptibilities and the gap equations, we calculated the critical temperatures and phases maps.

## S6. NUMERICAL PARAMETERS

In this section, the parameter sets used in this work are listed. For Fig. 2, basically $M = 4.25t_z$, $t_{xy} = 2.0t_z$, $\eta = t_z$, $\beta = 2.0t_z$, $\gamma = 1.0t_z$, and $\Delta_i = 0.05t_z$ are used. In Fig. 2(b), $n_1 = 0.05t_z$ for $\Delta_1'$, $\Delta_3$, and $\Delta_{41}$, and $n_1 = -0.05t_z$ for $\Delta_{42}$. In Fig. (c), $n_1 = 0.05t_z$ for $\Delta_1'$ and $\Delta_2$. $\beta = 1.0t_z$ , $\gamma = 2.0t_z$, and $n_1 = 0.01t_z$ for $\Delta_{41}$. $\beta = 2.0t_z$ , $\gamma = 1.0t_z$, and $n_1 = 0.01t_z$ for $\Delta_{42}$. In Fig. 2(d), $n_1 = n_2 = 0.05t_z$ for $\Delta_1'$. In Fig. 2(e), $n_1 = n_3 = 0.05t_z$ for $\Delta_1'$. $n_1 = n_3 = 0.01t_z$ for $\Delta_3$ and $\Delta_{41}$.

For Fig. 4, $M = 4.25t_z$, $t_{xy} = 2.0t_z$, $v = t_z$, $\beta = 2.0t_z$, $\gamma = 1.0t_z$, and $\Delta_i = 0.05$ are commonly used. In Fig. 4(e-h), $n_1 = 0.05$. In Fig. 4(i-l), $n_1 = n_3 = 0.05$.


[1] S. M. Young, S. Zaheer, J. C. Teo, C. L. Kane, E. J. Mele, and A. M. Rappe, Dirac semimetal in three dimensions, Physical Review Letters **108**, 140405 (2012).

[2] Z. Wang, H. Weng, Q. Wu, X. Dai, and Z. Fang, Three-dimensional dirac semimetal and quantum transport in $Cd_3As_2$, Physical Review B **88**, 125427 (2013).

[3] L. Aggarwal, A. Gaurav, G. S. Thakur, Z. Haque, A. K. Ganguli, and G. Sheet, Unconventional superconductivity at mesoscopic point contacts on the 3D Dirac semimetal $Cd_3As_2$, Nature Materials **15**, 32 (2016).

[4] L. He, Y. Jia, S. Zhang, X. Hong, C. Jin, and S. Li, Pressure-induced superconductivity in the three-dimensional topological dirac semimetal $Cd_3As_2$, NPJ Quantum Materials **1**, 1 (2016).

[5] H. Wang, H. Wang, H. Liu, H. Lu, W. Yang, S. Jia, X.-J. Liu, X. Xie, J. Wei, and J. Wang, Observation of superconductivity induced by a point contact on 3D Dirac semimetal $Cd_3As_2$ crystals, Nature Materials **15**, 38 (2016).

[6] L. M. Schoop, L. S. Xie, R. Chen, Q. D. Gibson, S. H. Lapidus, I. Kimchi, M. Hirschberger, N. Haldolaarachchige, M. N. Ali, C. A. Belvin, T. Liang, J. B. Neaton, N. P. Ong, A. Vishwanath, and R. J. Cava, Dirac metal to topological metal transition at a structural phase change in $Au_2Pb$ and prediction of $Z_2$ topology for the superconductor, Physical Review B **91**, 214517 (2015).

[7] K. W. Chen, D. Graf, T. Besara, A. Gallagher, N. Kikugawa, L. Balicas, T. Siegrist, A. Shekhter, and R. E. Baumbach, Temperature-pressure phase diagram of cubic Laves phase $Au_2Pb$, Physical Review B **93**, 045118 (2016).

[8] Y. Xing, H. Wang, C.-K. Li, X. Zhang, J. Liu, Y. Zhang, J. Luo, Z. Wang, Y. Wang, L. Ling, M. Tian, S. Jia, J. Feng, X.-J. Liu, J. Wei, and J. Wang, Superconductivity in topologically nontrivial material $Au_2Pb$, NPJ Quantum Materials **1**, 1 (2016).

[9] Y. Yu, Y. Xu, Y. Xing, J. Zhang, T. Ying, X. Hong, M. Wang, X. Zhang, S. Jia, J. Wang, *et al.*, Fully gapped superconducting state in $Au_2Pb$: A natural candidate for topological superconductor, Europhysics Letters **116**, 67002 (2017).

[10] J. Wu, Z. Feng, J. Wang, Q. Chen, C. Ding, T. Chen, Z. Guo, J. Wen, Y. Shi, D. Xing, *et al.*, Ground states of $Au_2Pb$ and pressure-enhanced superconductivity, Physical Review B **100**, 060103 (2019).

[11] A. P. Schnyder, S. Ryu, A. Furusaki, and A. W. Ludwig, Classification of topological insulators and superconductors in three spatial dimensions, Physical Review B **78**, 195125 (2008).

[12] M. Koshino, T. Morimoto, and M. Sato, Topological zero modes and dirac points protected by spatial symmetry and chiral symmetry, Physical Review B **90**, 115207 (2014).
[13] C.-K. Chiu, J. C. Teo, A. P. Schnyder, and S. Ryu, Classification of topological quantum matter with symmetries, Reviews of Modern Physics **88**, 035005 (2016).
[14] S. Kobayashi, K. Shiozaki, Y. Tanaka, and M. Sato, Topological blount's theorem of odd-parity superconductors, Physical Review B **90**, 024516 (2014).
[15] T. Hashimoto, S. Kobayashi, Y. Tanaka, and M. Sato, Superconductivity in doped dirac semimetals, Physical Review B **94**, 014510 (2016).
[16] S. Kobayashi and M. Sato, Topological superconductivity in dirac semimetals, Physical Review Letters **115**, 187001 (2015).
[17] A. S. Alexandrov, *Theory of superconductivity: from weak to strong coupling* (CRC Press, 2003).
[18] K.-H. Bennemann and J. B. Ketterson, *Superconductivity: Volume 1: Conventional and Unconventional Superconductors Volume 2: Novel Superconductors* (Springer Science & Business Media, 2008).
[19] L. Fu and E. Berg, Odd-parity topological superconductors: theory and application to $Cu_xBi_2Se_3$, Physical Review Letters **105**, 097001 (2010).
[20] S. Nakosai, Y. Tanaka, and N. Nagaosa, Topological superconductivity in bilayer rashba system, Physical Review Letters **108**, 147003 (2012).
